# Mechanisms of compensatory for cervical lordosis changes after laminectomy with fusion

**DOI:** 10.1186/s12893-022-01577-0

**Published:** 2022-04-07

**Authors:** Kai Yang, Xiang-Yu Li, Yu Wang, Chao Kong, Shi-Bao Lu

**Affiliations:** 1 Department of Orthopedics, Xuanwu Hospital, Capital Medical University, No.45 Changchun Street, Xicheng District, Beijing, China; 2 National Clinical Research Center for Geriatric Diseases, Beijing, China

**Keywords:** Laminectomy with fusion, Cervical sagittal alignment, Occiput-C2 angle, Cervical sagittal vertical axis, T1 slope

## Abstract

**Background:**

The compensatory mechanisms for cervical lordosis change after laminectomy with fusion was not clear. The objective of this study was to evaluate the compensatory behaviors for cervical lordosis change after laminectomy with fusion.

**Methods:**

This was a retrospective radiological analysis of 43 patients with cervical spondylotic myelopathy who underwent laminectomy with fusion (LCF). The following cervical parameters were measured: C2-7 Cobb angle (C2-7), occiput-C2 angle (O-C2), the cervical sagittal vertical axis (cSVA), and T1 slope (T1S). The difference was calculated for all angle parameters between the two time points using the following formula: the amount of change (Δ) = (value at the follow-up)—(preoperative value). Non-parametric tests and the t-test were used to compare the difference. The Pearson correlation test was performed, and stepwise multiple regression analysis was performed to determine the best correlation between ∆cSVA and ∆T1S.

**Results:**

The mean age of 43 patients was 65.51 ± 9.80 years. All patients were classified into two subgroups based on ΔcSVA: Group M (maintained) and, Group I (increased). The preoperative O-C2, C2-7, T1S, and cSVA were similar between Group M and group I (p = 0.950, p = 0.731, p = 0.372, and p = 0.152, respectively). Postoperative O-C2 and postoperative cSVA were significantly different (p = 0.036 and p = 0.004, respectively). ∆O-C2, ∆T1S and ∆cSVA were significantly different between the two groups (p = 0.006, p = 0.000, and p = 0.000, respectively). ΔcSVA had significant correlations with ΔO-C2 neutral angle (r = 0.377) and ΔT1S (r = 0.582). A linear regression equation was established: ΔcSVA = 0.602 + 0.103 * ΔT1S (R = 0.582, R^2^ = 0.339).

**Conclusions:**

The decrease of TIS should be the first and foremost compensation for the loss of lordosis in C2-7 segments after LCF. When the change of T1S alone can not prevent the deterioration of cervical sagittal balance, further increases in the O-C2 segment occur.

## Background

Cervical spondylotic myelopathy (CSM) is a common disease in older adults, that leads to cervical spinal cord function impairment. Surgery should be considered for patients who are refractory to conservative treatment. Laminectomy with fusion (LCF), a posterior decompression method, is a common procedure for multi-level CSM. However, C2-C7 Cobb (C2-7) angle reduction or cervical lordosis (CL) loss occurs commonly after LCF [1]. Even cervical positive sagittal malignment leading by severe CL loss is associated with poor outcomes [2].

To CL loss and cervical positive sagittal malignment, the cranial and caudal levels must compensate to maintain cervical sagittal balance. Nori et al. [3] studied patients with selective laminectomy and found that postoperative lower cervical kyphotic changes were compensated for by upper cervical lordotic changes. Ikeda et al. [4] found that the occipito-C2 (O-C2) angle increased and T1 slope (T1S) decreased as the compensatory mechanism for kyphotic change after anterior cervical corpectomy and fusion. Nevertheless, the compensatory mechanisms for cervical sagittal balance after LCF remain unclear to the best of our knowledge.

Therefore, in this study, we investigated the influence of O-C2 changes (ΔOC2) and T1S changes (ΔT1S) on the cervical sagittal balance after 4 or 5-level (C3-7 or C3-6) LCF to clarify the compensatory mechanisms.

## Patients and methods

### Patient population

The institutional review board of the authors’ institution approved this study. We retrospectively reviewed 110 patients who underwent LCF from January 2017 to February 2020. Patients were eligible if they met the following inclusion criteria: (1) age 18 years or older; (2) cervical cord compression in imaging findings; (3) at least one clinical sign of myelopathy; (4) four or five level decompression (C3-7 or C3-6) with LCF, and (5) at least 12 months of follow-up. Exclusion criteria were as follows: (1) McGregor line not clear; (2) preoperative cervical sagittal vertical axis (cSVA) > 40 mm; (3) malignancy; (4) neurological disorder; (5) post-traumatic myelopathy; (6) and history of cervical spine surgery. According to these criteria, 43 patients were included.

### Surgical procedure

After the induction of general endotracheal anesthesia, we installed Mayfield tongs, and then positioned the patient prone on an operating room table. We adjusted the Mayfield head holder to place the neck in a relatively extended position. An incision in the back of the neck was performed. Paraspinal muscles of patients were separated to expose the lamina. A high-speed burr was used to create a trough in the lamina on both sides. We then removed the lamina and spinous process. Lateral mass screws were inserted. We adjusted head holder to ensure cervical lordosis alignment. We then fixed cervical spine screws and rods system. Local bone autografts from the laminectomy were packed beneath and around the instrumentation.

### Radiological assessment

**Fig. 1:**
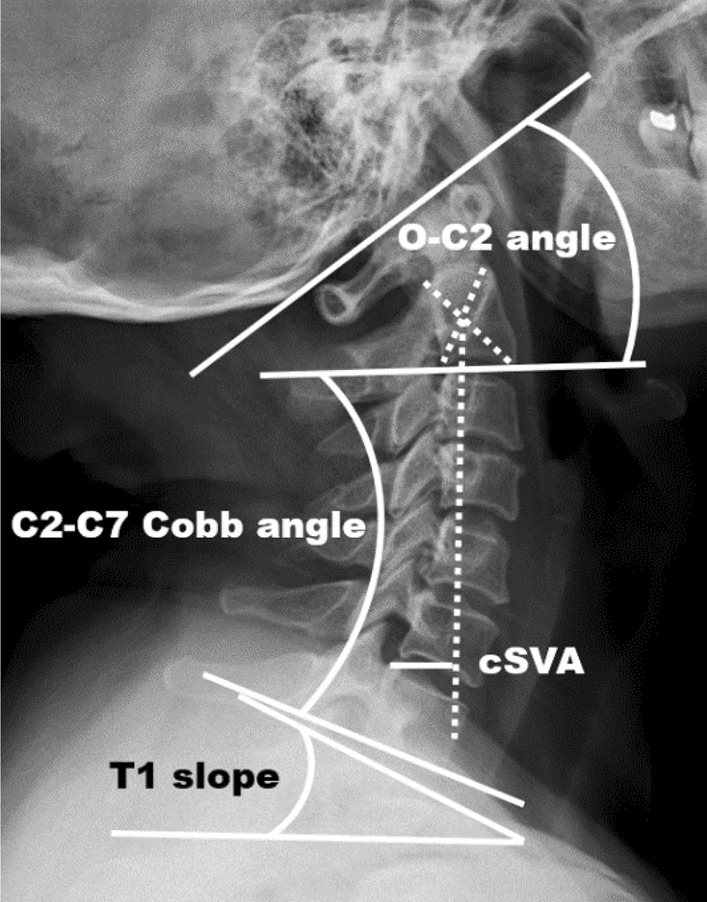
Measurements

Anteroposterior, lateral, flexion, and extension radiographs of the cervical spine were taken preoperatively. Lateral radiographs were taken postoperatively and at follow-up. Lateral radiographs of the cervical spine were taken with the patient in a comfortable standing position with the head facing forward for horizontal gaze. Cervical sagittal alignment parameters were measured by PACS system (Fig. 1). The O-C2 angle is the angle between the McGregor line and the inferior endplate line of C2. C2-7 angle is the angle between the C2 lower endplate and the C7 lower endplate. The cSVA is the distance from the posterosuperior corner of the C7 vertebral body to the vertical line from the center of the C2 vertebral body. T1S is the angle between the horizontal and superior endplate of the T1.

The difference was calculated for all angle parameters between the two time points using the following formula: the amount of change (Δ) = (value at follow-up)—(preoperative value).

**Fig. 2:**
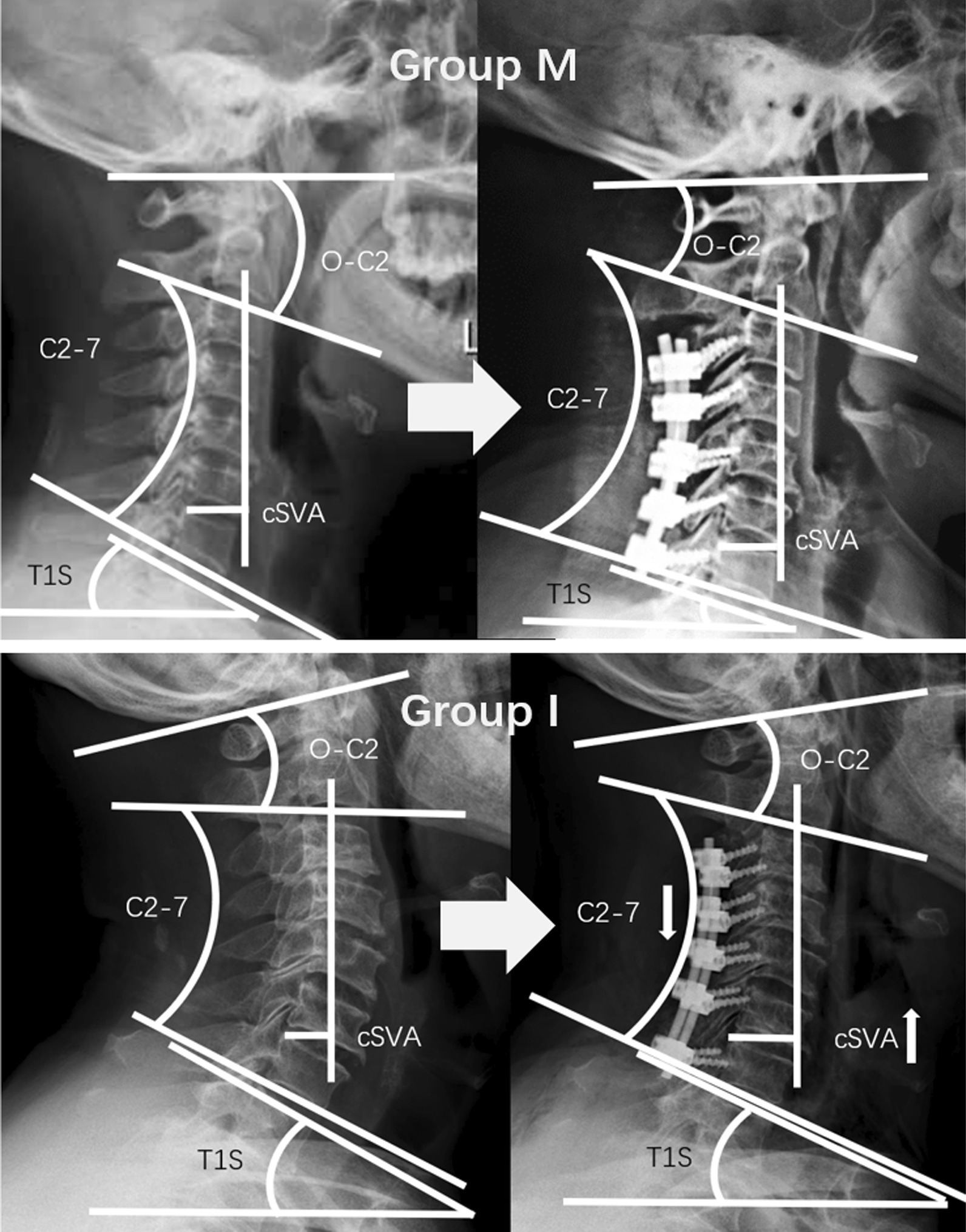
Group M and Group I

We classified all patients into two subgroups based on ΔcSVA: Group M (maintained), the cSVA value maintained or decreased after LCF surgery; Group I (increased), the cSVA value increased after LCF surgery (Fig. 2).

### Clinical assessment

Surgical outcome was assessed according to the recovery ratio in the modified JOA score (mJOA). Recovery rate (%) = (postoperative mJOA—preoperative mJOA) / (full score- preoperative mJOA) * 100%.

### Statistical analysis

Statistical analysis was performed using SPSS 24.0 software (IBM Corp, New York, USA). Measurement data were expressed in terms of mean ± standard deviation. The correlations between the parameters were analyzed using the Pearson correlation coefficient. Stepwise multiple linear regression was used to analyze independent variables that affect ΔSVA. The t-test was used to compare sagittal parameters and changes in radiographic measures before and after surgery. Nonparametric tests were used to analyze sex differences and surgery level differences. P < 0.05 was considered significant.

## Results

### Comparison between Group M and Group I

A total of 43 patients were enrolled, including 25 males and 18 females. The mean operation level was 4.30 ± 0.46. The mean follow-up period was 20.63 ± 6.53 months. The preoperative and postoperative parameters are shown in Table 1. There were no differences in age, gender, or follow up period between Group M and Group I (p = 0.841, p = 0.163, and p = 0.438, respectively). The preoperative parameters, including O-C2 neutral angle, C2-7 Cobb angle, T1S, and cSVA, were similar between Group M and Group I (p = 0.950, p = 0.731, p = 0.372, and p = 0.152, respectively). The postoperative O-C2 neutral angle and postoperative cSVA were significantly different between Group M and Group I (p = 0.036 and p = 0.004, respectively). There were no differences in preoperative mJOA and postoperative mJOA (p = 0.772 and p = 0.394, respectively).

**Table 1:** The preoperative and follow-up parameters in Group M and Group I

Characteristics	Total (43)	Group M (14)	Group I (29)	P value
*Age (years)*	65.51 ± 9.80	65.07 ± 7.96	65.72 ± 10.70	0.841
*Gender (Male/Female)*	25/18	6/8	19/10	0.163
*Follow up period (months)*	20.63 ± 6.53	19.50 ± 6.35	21.17 ± 6.66	0.438
*Numbers of levels operated (n)*	4.63 ± 0.49	4.43 ± 0.51	4.72 ± 0.45	0.062
*Operated level*				0.063
C3-6	16	8	8	
C3-7	27	6	21	
*Preoperative value*				
C2-7(degree)	14.99 ± 12.16	15.92 ± 11.76	14.54 ± 12.53	0.731
O-C2(degree)	26.87 ± 8.81	26.99 ± 7.80	26.81 ± 9.38	0.950
T1S(degree)	29.40 ± 8.59	31.49 ± 11.85	28.38 ± 6.51	0.372
cSVA(mm)	19.78 ± 9.94	22.92 ± 8.87	18.26 ± 10.21	0.152
mJOA	13.12 ± 1.1.52	13.21 ± 1.72	13.07 ± 1.44	0.772
*Final follow-up*				
C2-7(degree)	9.77 ± 11.06	10.99 ± 9.79	9.18 ± 11.74	0.622
O-C2(degree)	31.36 ± 7.82	27.80 ± 6.75	33.08 ± 7.82	**0.036**
T1S(degree)	26.62 ± 8.11	24.55 ± 8.74	27.61 ± 7.75	0.251
cSVA(mm)	22.94 ± 12.78	15.05 ± 11.72	26.74 ± 11.62	**0.004**
mJOA	16.60 ± 0.95	16.79 ± 0.89	16.52 ± 0.99	0.394

Bold value indicates *p* value < 0.05 and is statistically significant

### Changes of different parameters

The changes in parameters were compared between Groups M and I. Group I had a larger cSVA and O-C2 change than that of Group M (p < 0.001). However, Group M had a larger ΔTIS than Group I. ΔC2-7 was not significantly different between Groups D and I (p = 0.856). Although Group M had a better recovery rate than Group I, there was no significant difference (p = 0.504) (Table 2).

**Table 2:** The comparison of the changes of sagittal parameters and recovery rate between Group M and Group I

Parameters	Group M	Group I	P value
△C2-7(degree)	− 4.94 ± 6.48	− 5.36 ± 7.40	0.856
△O-C2(degree)	0.81 ± 6.19	6.27 ± 5.61	0.006
△T1S(degree)	− 6.94 ± 6.05	− 0.77 ± 4.23	0.000
△cSVA(mm)	− 7.87 ± 4.35	8.48 ± 7.07	0.000
Recovery rate (%)	74.49 ± 20.21	69.94 ± 20.94	0.504

### Correlation coefficient between sagittal parameters

ΔcSVA had significant correlations with ΔO-C2 neutral angle (r = 0.377) and ΔT1S (r = 0.582). However, ΔcSVA did not correlate with ΔC2-7. ΔO-C2 had a negative correlation with ΔC2-7 (Table 3).

**Table 3:** Correlation analysis between different change parameters

Characteristics	△O-C2	△C2-7	△T1S	△cSVA
Age	0.165 0.290	0.158 0.311	− 0.137 0.381	− 0.081 0.606
△O-C2		− **0.363** **0.017**	0.081 0.608	**0.377** **0.013**
△C2-7			0.184 0.239	− 0.268 0.083
△T1S				**0.582** **0.000**

Bold value indicates *p* value < 0.05 and is statistically significant

### Stepwise multiple linear regression analysis for ΔcSVA

The stepwise multiple linear regression analysis was used to model the relationship between ΔcSVA and potential factors. A linear regression equation was established: ΔcSVA = 0.602 + 0.103 * ΔT1S (R = 0.582, R^2^ = 0.339). (As shown in Tables 4 and 5).

**Table 4:** Stepwise multiple regression analysis

Model	R	R^2^	Adjusted R^2^	Standard error of estimate	R^2^ change	F change	Sig. F change
1	0.582	0.339	0.323	0.820	0.339	21.037	0.000

The predictor variable is △T1S

The dependent variable is △cSVA

**Table 5:** The coefficient and constant of the linear regression equation

Model		Regression coefficient	Standard deviation	Standardized coefficient	t value	P value	Tolerance	VIF
1	Constant	0.602	0.140		4.307	0.000		
	△T1S	0.103	0.022	0.582	4.587	0.000	1.000	1.000

*VIF* variance inflation factor

## Discussion

There was a tendency of decreased CL in patients who underwent the posterior LCF with CSM. Several authors previously demonstrated loss of CL following a posterior surgical approach. Roguski et al. [5] found that patients on average lost 4.2 degrees of lordosis and had a mean postoperative Cobb angle of − 3.4 ± 16.3 degrees. Cabraja et al. [6] found that patients lost an average of 6.5 degrees of lordosis, with a mean postoperative C2-7 of − 6.6 ± 13.3 degrees. Lee et al. [7] found that a mean C2-7 changed from − 10 ± 11.6 degrees preoperatively to − 5.1 ± 12.0 postoperatively after extensive laminectomy with fusion. In the present study, C2-7 significantly decreased in Groups D and I. The average postoperative change of the C2–C7 was − 4.94 ± 6.48° in Group M, − 5.36 ± 7.40° in Group I.

Although cervical lordosis changed after laminectomy, CL was not the main parameter to analyze the cervical sagittal balance [8]; cSVA is an important parameter associated with clinical symptoms and used evaluate cervical balance [2, 9]. To maintain the sagittal balance of the cervical spine, compensatory behaviors were maintained in the cSVA in Group M. For patients with increased cSVA, three patients had postoperative cSVA > 40 mm. Three patients had relatively poor outcomes with recovery rates no more than 60%. There was no significant difference in recovery rate between Groups I and D. We believe that maintaining cervical sagittal balance is critical for achieving excellent clinical outcomes. Sielatycki et al. [9] found that patients with kyphosis cervical spine who underwent LCF had improved clinical outcomes associated with creating more lordosis and decreasing SVA. However, they did not find such an association in patients with lordosis and stated that any amount of lordosis might be sufficient for LCF.

Lee et al. [10] introduced the term T1 slope in 2012. Since then, studies have concentrated on the preoperative relationship between T1S and CL to predict the postoperative change of CL [11, 12]. Other studies demonstrated that the T1 (C7) slope was associated with factors of cervical sagittal alignment, including C2–C7 lordosis and C2–C7 SVA [13, 14]. Kennamer et al. [15] in a recent study of cervical alignment with posterior cervical fusions, demonstrated a relationship between increased SVA and increasing T1 slope and worsened clinical outcomes. Hyun et al. [11] obtained a similar result, and the authors suggested that this phenomenon could indicate a compensatory mechanism to regulate the angle of gaze. Thus, when the cSVA changes, a corrective change in T1S ensues. Nevertheless, it is unknown whether a separate change of T1S was sufficient to affect cSVA, especially under the condition of the loss of cervical lordosis. The T1 vertebra is the foundation of the cervical spine. Because C7/T1 is a transition zone, this level is exposed to unique biomechanical forces, particularly flexion or translation stress. When the C2-7 changes, T1S can be altered by changing thoracic kyphosis or other factors to adjust cervical sagittal balance and preserve horizontal gaze [16, 17]. Hyun et al. [11] showed a mean T1S change from 25.7 ± 6.9 degrees preoperatively to 23.2 ± 8.0 degrees postoperatively. In the present study, in Group M, ∆TIS was significantly smaller than Group I (− 6.94 ± 6.05°, − 0.77 ± 4.23° respectively). Thus, in Group M, but not in Group I, an increase in cSVA was avoided by a sufficient reduction in TIS. Using stepwise multiple linear regression analysis, we found that ΔcSVA was affected primarily by ∆TIS. Therefore, the decrease of TIS should be the first and foremost compensation for the change of cSVA and the loss of C2-7. When the T1S could not decrease sufficiently or increase, compensation could not be achieved. Next, O-C2 change could participate in compensation to achieve gaze horizon. Li et al. [18] studied patients with lumbar degenerative disease and found that thoracic hypokyphosis compensation prevented T1 slope increase; thus, T1S change was related to thoracic kyphosis change. However, patients without sufficient T1S compensation might have reasons that limit thoracic kyphosis change, including spondylitis, diffuse idiopathic skeletal hyperostosis, sarcopenia, and debility, and these patients are challenging to maintain cervical sagittal alignment balance.

Correlation analysis revealed a negative correlation between the C2–7 and ΔO-C2 neutral angle postoperative changes. In the present study, postoperative O-C2 angle and ΔO-C2 angle in Group I were significantly larger than in Group M. This finding suggests that the O-C2 increased to compensate for the balance adjustment and horizontal gaze in Group I. In addition, there was a significant positive correlation between ∆cSVA and ∆O-C2. As the ∆cSVA increased, so did the ∆O-C2. Thus, the forward tilt of the C2–C7 segments could be compensated for by the hyperextension of the O-C2 segments after LCF to insure horizontal gaze. Woodroffe et al. [19] found that the inclusion of C2 in the fusion construct resulted in increased sagittal balance, increasing the SVA and T1S. This finding indicated that when the O-C2 change was eliminated, the cervical sagittal balance would be affected. According to this analysis, the O-C2 change was the compensatory mechanism for adjusting cervical sagittal balance after LCF when TIS change would not preserve or reduce the original cSVA.

**Fig. 3:**
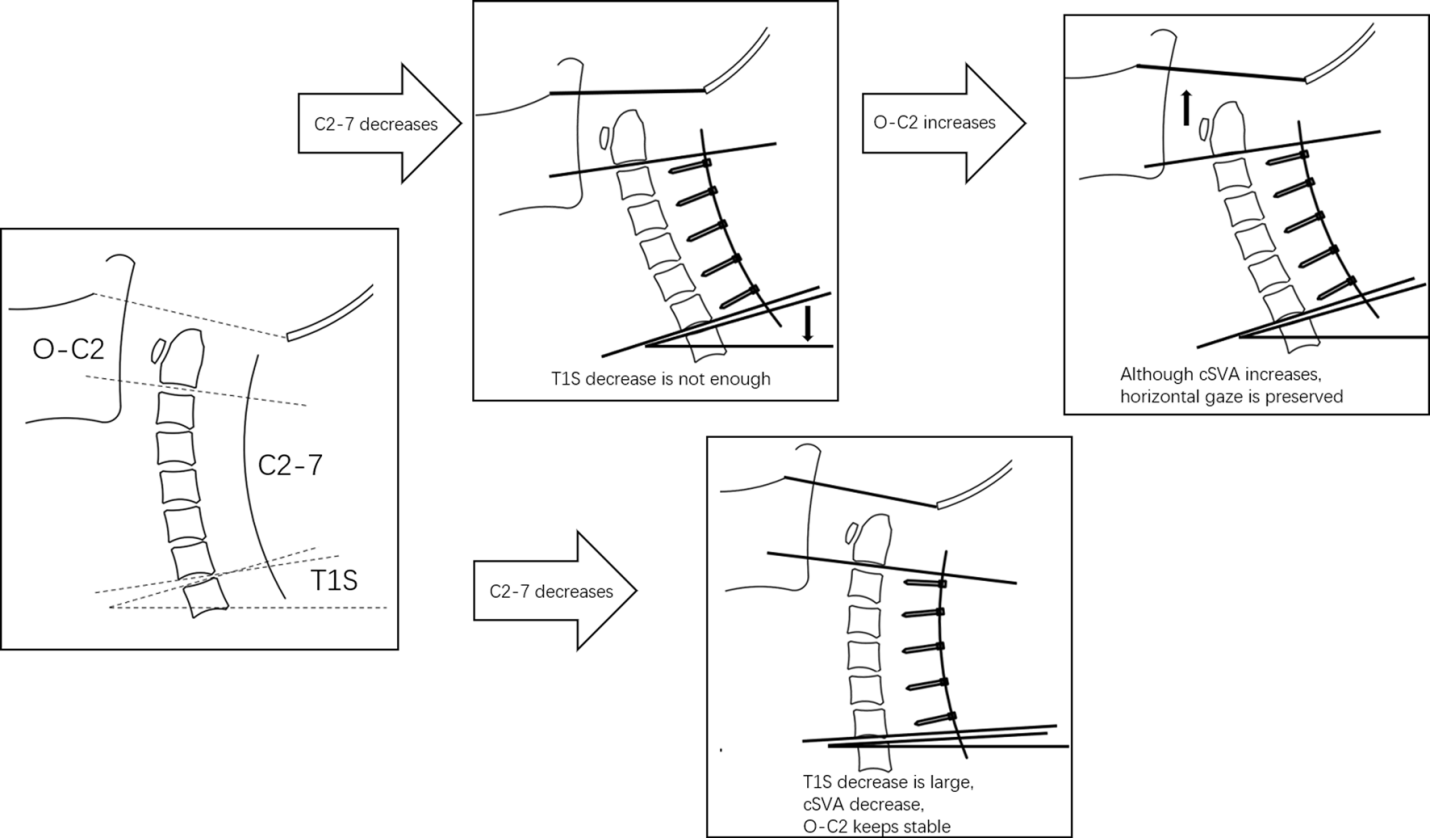
Schema describing the various compensatory mechanisms after laminectomy with fusion

We summarized the compensatory mechanisms in Fig. 3. O-C2 angle and T1S would compensate for loss of CL. O-C2 compensation is the supplement of T1S compensation in cervical alignment balance adjustment.

This was a retrospective cohort study; thus, several limitations should be noted. First, the number of cases in this study was small, especially in Group M, and there were not many cases to confirm our findings. Second, the level of LCF was not uniform. Third, the radiographic follow-up time was relatively short. Finally, this study was a single-center follow-up analysis; future studies should include several centers.

## Conclusions

The decrease of TIS should be the first and foremost compensation for the loss of lordosis in C2-7 segments after the posterior laminectomy with fusion. When the change of T1S alone could not prevent the deterioration of cervical sagittal balance, further increase in the O-C2 segment happened.

## Data Availability

In attempt to preserve the privacy of patients, clinical data of patients will not be shared; data can be available from the corresponding author upon request.

## Abbreviations

LCF: Laminectomy with fusion
CSM: Cervical spondylotic myelopathy
O-C2: Occiput-C2
C2-7: C2-7 Cobb angle
T1S: T1 slope
CL: Cervical lordosis
cSVA: Cervical sagittal vertical axis
